# Pericatheter urethrogram after anastomotic urethroplasty: Is it a must?

**DOI:** 10.12669/pjms.345.15266

**Published:** 2018

**Authors:** Ali Haider, Syed Mamun Mahmud

**Affiliations:** 1 Ali Haider, South Asian Institute of Urology and Nephrology (SAIUN), A Unit NSM Health Care, Suite 603, 6th Floor, Alkhaleej Tower, Shaheed-e-Millat Road, Karachi Pakistan; 2 Syed Mamun Mahmud, Lifecare Hospital, HOD, Department of Urology, Post Code 133500, Abu Dhabi, UAE

**Keywords:** Anastomotic Urethroplasty and Follow Up, Pericatheter Urethrogram

## Abstract

**Objective:**

To share our initial experience of patient undergoing anastomotic Urethroplasty and trial without catheter, without post Urethroplasty pericatheter urethrogram.

**Methods:**

Prospectively maintained records of all patients undergoing standard transecting anastomotic Urethroplasty by single surgeon (one of the authors) at The Kidney Centre PGTI Karachi, Pakistan and Lifecare Hospital Abu Dhabi UAE from September 2006 to December 2017 were reviewed. In all except two cases, supra pubic catheter was removed at 2^nd^ weeks and per urethral catheter by 4 to 5 weeks following which patients were assessed for TWOC without pericatheter urethrogram. Patients were further advised to follow up with Uroflowmetry (UFM) at one week, one month, three and 12 months. In our series, Qmax less than 15 ml/s on UFM were considered to have recurrence and these patients were subjected to ascending urethrogram after six weeks of procedure.

**Results:**

There were 18 patients who underwent anastomotic Urethroplasty in bulbar urethra. The mean age of study patients was 37.2+11.2 years with p-value of 0.84. The recurrence rate of urethral stricture was 16.6 % (3/18 patient) with Qmax of 4.6 and 7.2ml/sec with mean follow-up period of 13.82+13.4 months (range 3-53 months) 02 patients developed infection. No patient developed incontinence or impotence.

**Conclusion:**

We found pericatheter urethrogram is not mandatory as a routine for all tension free anastomotic Urethroplasty before per urethral catheter removal. However, it may have a role in difficult cases with tension anastomoses or re-do procedure. This will avoid risk of infection, radiation exposure and extra cost.

## INTRODUCTION

Urethroplasty is one of the oldest procedures with established results. It is interesting to note that there is still great variation among urologist for more than half century old procedure^1^ to evaluate and follow patients postoperatively for restricturing.^2^-^5^ The recurrence of urethral stricture is also not clearly defined for post urethroplasty patients. However some centers considered Qmax less than 10ml/second whereas some as less than 15ml/sec on UFM as recurrence.^5^,^6^ Similarly, there is no standardization to follow patients in immediate course. A survey was conducted between July 2010 and October 2010 internationally under American Urological Association (AUA) to appraise Urologists’ practice in following patients after Urethroplasty. The survey was widely participated by practicing urologists from North & South America and Europe and was published in The Journal of Urology in 2011. It was observed that about 10% of practicing urologists was not conducting pericatheter urethrogram before trial of void to assess for anastomotic leak.^3^

Thus, our study may serve as a pilot study to assess the role of post urethroplasty pericatheter urethrogram before trial without catheter (TWOC) in patients undergoing anastomotic urethroplasty.

## METHODS

Prospectively maintained records of all patients undergoing standard transecting anastomotic urethroplasty by single surgeon (one of the authors) at The Kidney Centre PGTI Karachi, Pakistan and Lifecare Hospital Abu Dhabi UAE from September 2006 to December 2017 were reviewed. Among these cases, some patients already had suprapubic catheter and rest had it peroperatively. All procedures were done transperineally except initial two cases where combined abdomen-perineal approach was adopted. In all except two cases, supra pubic catheter was removed at 2^nd^ weeks and per urethral catheter by 4 to 5 weeks following which patients were assessed for TWOC without pericatheter urethrogram. Patients were further advised to follow up with Uroflowmetry (UFM) at one week, one month, three and 12 months. In our series, Qmax less than 15 ml/s on UFM were considered to have recurrence and these patients were subjected to ascending urethrogram after six weeks of procedure. Incontinence was defined as no pads used to protect against urinary leakage.^7^ Erectile function were only assessed by patients reporting intercourse with vaginal penetration. Those not achieving this were identified as impotent.^7^

## RESULTS

There were 18 patients who underwent anastomotic urethroplasty in bulbar urethra. The mean age of study patients was 37.2±11.2 years with p-value of 0.84. One patient lost to follow up after first OPD visit following successful void after catheter removal. One patient was excluded as he had neurogenic bladder with unilateral reflux which might affect his voiding function. The recurrence rate of urethral stricture was 16.6 % (3/18 patient) with Qmax of 4.6 and 7.2ml/sec. 02 patients developed infection. No patient developed incontinence or impotence.

**Table-I:** Results of Urethroplasty.

	Mamun et al.	Mundy et al.	Jenkins et al.
No. of patients	18	82	73
Mean follow up (months)	13.82	180	60
Restricture	16.6%	14%	20%
Complications	11.11%	7%	15%

There was no urethral fistula. The mean follow-up of participants was 13.82±13.4 months (range 3-53 months). The mean Qmax on UFM of study patients was 31.2±18.3 (range 15.3 -53.1 ml/s) with p-value of <0.01.

## DISCUSSION

Currently, in the developed world, majority strictures are relatively short and are situated in the bulbar urethra. There is significant evidence that these are best treated by excision and end-to-end anastomosis if they are short enough or by patch Urethroplasty using a buccal mucosal graft if they are longer. A tension free anastomosis is the key to success for End to End Anastomotic Urethroplasty.^8^ Substitution Urethroplasty originally used skin grafts or flaps to restore urethral caliber and was technically demanding.^9^,^10^ Over the last five decades as the Urethroplasty is being developed^1^, an aesthetic care, antibiotic treatment, and improvements in anatomic understanding and technique have made the procedure safer and outcome more promising. In fact, recent assessment of the cost-effectiveness of urethroplasty compared with dilatation and urethrotomy suggests that there is no advantage of doing more than one urethrotomy before proceeding to urethroplasty^11^,^12^ and if a patient has a significant stricture, then a primary urethroplasty is certainly the best treatment option.^11^ Majority of centres around the world follow patients at three weeks postoperatively with a pericatheter urethrogram. However, as we found in survey under American Urological Association (AUA) that about 10% of practicing urologists are not performing pericatheter urethrogram before trial of void.^3^ Another study reported from The University of Texas South Western Medical Centre by Morey et al shared their concerns on performing pericatheter Urethrogram as a routine in anastomotic Urethroplasty. In the study, authors suggested that pericatheter urethrogram can be omitted in cases of tension free End to End Anastomotic Urethroplasty. They reported that performance of pericatheter urethrogram resulted in causing pain as well as disruption of biofilm (mucopurulent membrane) along the urethral catheter as well as chances of infection around urethral anastomosis as these organism are much more resistant to antibiotics and can result in deep seated abscesses, anastomotic failures and urethrocutaneous fistula formation. Besides there has also been concerns regarding exposure to radiation and additional cost.^2^ On the other hand Uroflowmetry (UFM) a completely non-invasive test has been a common method to evaluate men with urethral stricture.^13^-^15^ The test gives three objective data points of maximum flow rate (Qmax), average flow rate and Voided Volume (VV). Most UFM equipment will also generate a voiding curve, depicting flow rate over time. The data most often used is the Qmax values.^16^ The commonly cited cut point of 15 ml per second been used to screen for recurrence of urethral stricture in majority of citations.^6^ There are other factors showing more usefulness in diagnosing stricture and predicting its recurrence, including the shape of the voiding curve and the presence of voiding symptoms. However, we acknowledge that static UFM data points are influenced by factors other than the degree of urethral obstruction, including inherent bladder contractility, patient age, history of pelvic/bladder surgery, comorbidities like diabetes and the degree of obstruction from prostatic hypertrophy. Taking all this in to account, we reviewed our data for the patients who underwent anastomotic Urethroplasty and studied the role of tools other than pericatheter urethrogram in tension free anastomotic Urethroplasty. We found UFM a very useful tool along with symptoms evaluation, for the patient who underwent tension free anastomotic Urethroplasty. We understand the limitation of small sample size with shorter follow up for half a century old procedure. However our results are reasonably comparable to related work reported in the world literature. Thus we believe despite small sample size, our study may serve as a pilot study in generating stronger evidence in terms of larger scale Randomized control trials in high volume centers of excellence and to decide to rebel traditionalism or to stick to old pathways through more scientific approach.

## CONCLUSION

We found pericatheter urethrogram is not mandatory as a routine for all tension free anastomotic urethroplasty before per urethral catheter removal. However, it may have a role in difficult cases with tension anastomoses or re-do procedure. This will avoid risk of infection, radiation exposure and extra cost. We are in early phase of our study and for stronger evidence; randomized trials with larger sample size are required.
